# The genetic difference between Western and Chinese urothelial cell carcinomas: infrequent *FGFR3* mutation in Han Chinese patients

**DOI:** 10.18632/oncotarget.8404

**Published:** 2016-03-26

**Authors:** Xiaotian Yuan, Cheng Liu, Kun Wang, Li Liu, Tiantian Liu, Nan Ge, Feng Kong, Liu Yang, Magnus Björkholm, Yidong Fan, Shengtian Zhao, Dawei Xu

**Affiliations:** ^1^ Department of Central Research Laboratory and Urology, Shandong University Second Hospital, Jinan, China; ^2^ Department of Medicine, Division of Haematology and Centre for Molecular Medicine (CMM), Karolinska University Hospital Solna and Karolinska Institutet, Stockholm, Sweden; ^3^ Karolinska Institutet-Shandong University Collaborative Laboratory for Cancer Research, Jinan, China; ^4^ Department of Urology, Shandong University Qilu Hospital, Jinan, China; ^5^ Shandong University Nursing School, Jinan, China; ^6^ Department of Pathology, Shandong University School of Medicine, Jinan, China

**Keywords:** FGFR3 mutation, racial disparity, TERT promoter mutation, urothelial bladder carcinoma, upper track urothelial carcinoma

## Abstract

Urothelial cell carcinoma (UCC) includes urothelial bladder carcinoma (UBC), renal pelvic carcinoma (RPC) and ureter carcinoma (UC), and its incidence varies dependent on geographical areas and tumor locations, which indicates different oncogenic mechanisms and/or different genetic susceptibility/environment exposure. The activating mutations of the *fibroblast growth factor receptor 3* (*FGFR3*) gene and telomerase reverse transcriptase (TERT) promoter are the most frequent genetic events in UCCs. These mutations have clinical utilities in UCC initial diagnostics, prognosis, recurrence monitoring and management. However, the vast majority of the results are obtained from studies of UCC patients in Western countries, and little has been known about these in Han Chinese patients. In the present study, we screened the *FGFR3* gene and TERT promoter for mutations in 116 UBC, 91 RPC and 115 UC tumors from Han Chinese patients by using Sanger Sequencing. TERT promoter mutations occurred at a high frequency in these UCC patients, comparable with that seen in Western patients, however, the *FGFR3* mutation was surprisingly lower, only 9.4% for UBCs, 8.8% for RPCs and 2.6% for UCs, respectively. Taken together, the *FGFR3* gene is an infrequent target in the pathogenesis of Han Chinese UCCs, and its mutation detection and targeted therapy have limited clinical utility in these patients. Our results underscore the need for extensive characterization of cancer genomes from diverse patient populations, thereby contributing to precision medicine for cancer treatment and prevention.

## INTRODUCTION

Urothelial cell carcinomas (UCCs) are originated from urothelium, and the vast majority of UCCs occur in the bladder (urothelial bladder carcinoma, UBC), whereas < 10% of them are located at the ureter or the renal pelvis [ureter carcinoma (UC), or renal pelvic carcinoma (RPC)] which are collectively called as upper tract urothelial carcinomas (UTUCs) [1–3]. There are different genetic and epigenetic alterations and different pathogenic pathways in UCCs at different anatomical locations, but certain oncogenic mutations do occur in all the three subgroups [1, 2, 4]. For instance, telomerase reverse transcriptase (TERT) promoter and *fibroblast growth factor receptor 3* (*FGFR3*) gene mutations are not only the most frequent genetic events in UBCs, but also widespread in UTUCs including both RPC and UC [3, 5–13]. In particular, 60 to 80% of non-muscle-invasive UBCs harbor *FGFR3* mutations and the mutations are similarly associated with low-stage or non-invasive UTUCs [3, 4, 14]. TERT promoter mutations are associated with the activation of telomerase, an RNA-dependent DNA polymerase essential to malignant transformation [15–18], while FGFR3 is a tyrosine kinase receptor that mediates the effects of fibroblast growth factors (FGFs) and stimulates the RAS-mitogen-activated protein kinase (MAPK) and phosphatidylinositide-3 kinase–AKT pathway [4, 14, 19]. The accumulated evidence has suggested that the mutant FGFR3 functions as an oncogenic driver in the development of UCCs [4, 14, 19, 20]. Moreover, the mutations have been shown as useful clinical biomarkers for UCC outcome prediction, diagnostics and recurrence monitoring, and inhibiting FGFR3 for therapeutic purpose is under development [1, 3, 4, 14, 21, 22].

The above observations are mainly obtained from the analyses of UCC patients in Western or European countries, however, little is known about the *FGFR3* mutation status in Han Chinese patients with UCCs. In has been well characterized that there is a racial difference in genetic alterations in other types of cancer [23]. In the present study, we thus determine whether this is the case in UCCs. By comparing the frequency of the *FGFR3* and TERT promoter mutations between Chinese patients and those from European countries or USA, we surprisingly found a much lower prevalence of *FGFR3* mutations (< 10%) in the analyzed Chinese patients compared to that found in UCC patients from Western countries [1, 3, 14, 21]. Our results strongly indicate that the oncogenic pathway underlying the development of UCCs may differ between Han Chinese and Western patients; the very low prevalence of the *FGFR3* mutation implies a limited clinical utility of its detection for Han Chinese UCCs.

## RESULTS

### Patient characteristics

Forty-one of patients were excluded due to the unsuccessful amplification of FGFR3 exon 7 (most cases) and/or exon 15 (a few of them) for sequencing analyses. Thus, a total of 91 RPC, 115 UC and 116 UBC patients whose FGFR3 sequences were evaluable were included. Tumour grading and staging were performed according to the criteria of the World Health Organization and the TNM classification of the International Union Against Cancer (2002). Patient clinical characteristics, including sex, age at diagnosis, tumor size and other histo-pathological characteristics, and metastases, are summarized in Tables 1–3.

**Table 1:** *FGFR3* mutations in relation to clinical and tumor characteristics in patients with renal pelvis carcinoma (RPC)

Variable informative cases (*n* = )	*FGFR3* mutation		*P-*value
	Mutated (*n* = 8)	wild-type (*n*= 83)	
*Age at diagnosis* (n= 91)			
Mean years (Mean ± SD)	62 ± 6.302	63.17 ± 11.084	0.77 (ns)*
Median (range) years	63 (53−67)	64 (36−85)	
*Gender* (n = 91)			0.721 (ns)
Female	3	39	
Male	5	44	
*TNM stage*(n = 91)			0.248 (ns)
pTa + pT1	2	9	
≥ pT2	6	74	
*Pathology stage* (n = 91)			0.108 (ns)
G1	0	0	
G2	4	19	
G3	4	64	
*Tumor size*(n = 86)			0.099 (ns)
< 3 cm	0	26	
≥ 3 cm	8	52	
*Distant metastases*(n = 91)			0.059 (ns)
Yes	2	3	
No	6	80	
*Lymph node metastases (n = 91)*			0.375 (ns)
Yes	1	4	
No	7	79	
*TERT promoter mutation (n = 91)*			0.721 (ns)
Yes	4	35	
No	4	48	

* ns = not statistically significant.

**Table 2:** *FGFR3* mutations in relation to clinical and tumor characteristics in patients with ureter carcinoma (UC)

Variable informative cases (*n* = )	*FGFR3* promoter mutation		*P-*value
	Mutated (*n* = 3)	wild-type (*n*= 112)	
*Age at diagnosis* (n= 115)			
Mean years (Mean ± SD)	67 ± 5	66.44 ± 9.978	0.922 (ns)*
Median (range) years	67 (62−72)	67 (32−87)	
*Gender* (n = 115)			1.00 (ns)
Female	1	40	
Male	2	72	
*TNM stage*(n = 115)			1.00 (ns)
pTa + pT1	0	28	
≥ pT2	3	84	
*Pathology stage* (n = 115)			0.147 (ns)
G1	0	0	
G2	2	26	
G3	1	86	
*Tumor size*(n = 94)			0.598 (ns)
< 3 cm	1	49	
≥ 3 cm	2	42	
*Distant metastases*(n = 115)			1.00 (ns)
Yes	0	5	
No	3	107	
*Lymph node metastases*(n = 115)			1.00 (ns)
Yes	0	8	
No	3	104	

* ns = not statistically significant.

**Table 3:** *FGFR3* mutations in relation to clinical and tumor characteristics in patients with urothelial bladder carcinoma (UBC)

Variable informative cases (*n* = )	*FGFR3* mutation		*P-*value
	Mutated (*n* = 11)	wild-type (*n*= 105)	
*Age at diagnosis* (n= 116)			
Mean years (Mean ± SD)	59 ± 12	64 ± 11	0.172 (ns)*
Median (range) years	54 (41−78)	66 (21−87)	
*Gender* (n = 116)			0.739 (ns)
Female	2	18	
Male	9	87	
*TNM stage*(n = 116)			0.122 (ns)
pTa + pT1	6	84	
≥ pT2	5	21	
*Pathology stage* (n = 116)			0.108 (ns)
*G1*	3	11	
G2	1	42	
G3	7	52	
*Tumor size*(n = 107)			0.787 (ns)
< 3 cm	5	60	
≥ 3 cm	3	39	
*Distant metastases*(n = 116)			0.667 (ns)
Yes	0	3	
No	11	102	
*Lymph node metastases (n = 116)*			0.590 (ns)
Yes	1	6	
No	10	78	
*TERT promoter mutation (n = 116)*			0.451 (ns)
Yes	4	56	
No	7	49	

* ns = not statistically significant.

### Infrequent FGFR3 mutations in Han Chinese RPC and UC tumors

**Figure 1:**
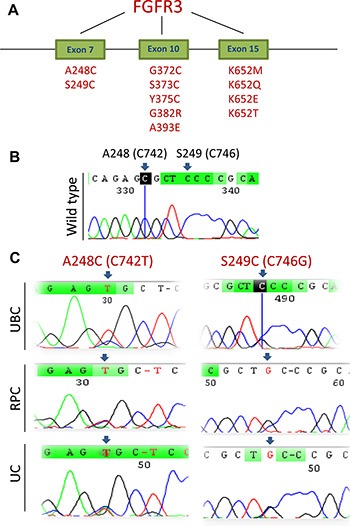
*FGFR3* mutations identified in UCC tumors (**A**) The schematic illustration of frequently mutated positions at the *FGFR3* gene. (**B**) The wild type of the *FGFR3* sequence at C742 and C746 in a UCC tumor, as shown in Sanger sequencing chromatographs. (**C**) Sequencing chromatographs of the *FGFR3* locus in tumor genomic DNA from six UCC patients obtained by Sanger sequencing. Shown are A248C (C742T) and S249C (C746G) mutations detected in six tumours (two UBCs, two RPCs and two UCs). Of note, the UBC tumor with C746G mutation (C, top right panel) was heterozygous, and the reading was still C, but a G signal was present.

We analysed the *FGFR3* exons 7, 10 and 15 in tumor DNA derived from evaluable 91 RPC patients. The mutation was found in 8 of 91 (8.8%) RPC tumors (Figure 1A–1C and Table 1). Eight FGFR3-mutation-carrying RPC tumours included two with A248C, 4 S249C, 1 G372C, and 1 T375C. Two of 11 patients (18%) with tumor stage pTa/T1 while 6 of 82 (7%) in stage ≥ pT2 were positive for the mutation, a difference which was not statistically significant (*P* = 0.24) (Table 1). In addition the mutation was not correlated with patient age, sex, and tumor sizes (Table 1).

**Figure 2:**
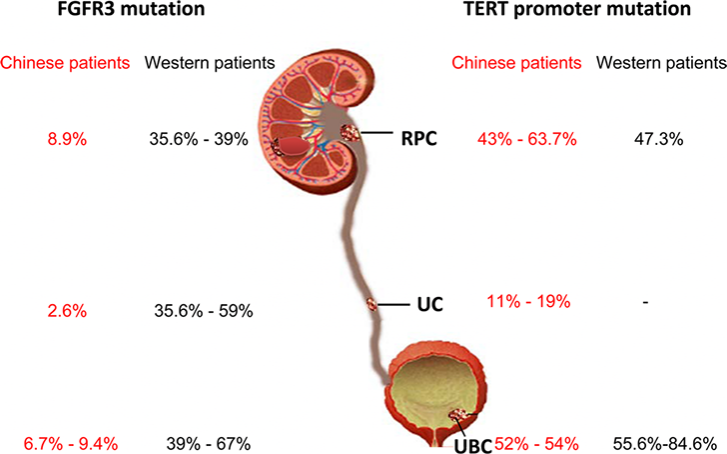
Differences in FGFR3 gene and TERT promoter mutations between Han Chinese and Western patients with urothelial cell carcinoma RPC, renal pelvic carcinoma; UC, ureter carcinoma; UBC, urothelial bladder carcinoma. -: No data available from Western UC patients.

The same analysis was performed on tumors from 115 UC patients and we identified 3 (2.6%) of UC tumors with the *FGFR3* mutation, even lower than that recorded in RPC tumors (Figure 1 and Table 2). Three mutant FGFR3 tumors included 1 A248C, 1 S249C, and 1 L652G. All the 3 tumors were from patients with > pT2 stage and there was no correlation between the mutation and other clinical-pathological variables including age, sex, or tumor sizes (Table 2, Figure 2).

### A lower prevalence of the FGFR3 mutation in Han Chinese UBC tumors

We further determined the *FGFR3* mutation in tumors from Han Chinese UBC patients. The *FGFR3* mutations were identified in 11 of 116 evaluable tumors (9.4%) and A248C, S249C, G372C and L652G were observed in 2, 3, 3 and 3 tumors, respectively (Figure 1A–1C and Table 3). Interestingly, one tumor harbored both S249C and G372C mutations. The stage distribution of the patients carrying these mutations was 4 in pTa, 2 in pT1, 1 in pT2, 1 in pT3 and 2 in pT4, respectively. The presence of the mutation was not predicted by age, sex, tumor sizes, stage or grade (Table 3, Figure 2).

### Stage and grade comparison between the present cohort of Han Chinese patients and that reported in European patients

The *FGFR3* mutation is more prevalent in pTa/pT1 stages of UCC. Thus, a critical issue is to make sure that the low rate of the *FGFR3* mutation found in the present cohort of patients is truly representative rather than due to the skewness in the distribution according to clinical stage. For this purpose, we compared our cohort of patients with a published report by van Oer et al. in which the *FGFR3* mutation in European patients with UBC, RPC and UC analysed simultaneously [3]. Table 4 reveals a highly significant lower rate of *FGFR3* mutations in the present Chinese UBC patient group despite the vast majority of them being in stage pTa/T1 compared to patients in van Oers’ cohort. An unexpected difference in the *FGFR3* mutation was similarly observed between Chinese and European patients with RPC and UC, although the stage/grade distribution also significantly differed in these two cohorts (Table 4, Figure 2).

**Table 4:** Comparison of UCC patient and tumor characteristics in the present study and findings of van Oer et al.*

Variable informative cases (*n* = )	Present study	van Oers et al.	*P* value
**UBC**	***n* = 116 (100%)**	***n* = 105 (100%)**	**< 0.001**
*FGFR3 mutation*	11 (9.4)	48 (46)	
*Tumor stage*			**0.031**
pTa + pT1	90 (78)	70 (60)	
≥ pT2	26 (22)	47 (40)	
*Histologic grade*			
G1	14 (12)	21 (18)	0,947
G2	43 (37)	36 (31)	
G3	59 (51)	60 (51)	
**RPC**	**91 (100%)**	**80 (100%)**	
*FGFR3 mutation*	8 (8.8)	31 (39)	**< 0.001**
*Tumor stage*			**0.002**
pTa + pT1	11 (12)	26 (33)	
≥ pT2	80 (88)	54 (67)	
*Histologic grade*			
G1	0	9 (11)	
G2	23 (27)	37 (46)	
G3	68 (73)	34 (44)	
**UC**	**115 (100%)**	**63 (100%)**	
*FGFR3 mutation*	3 (2.6%)	37 (59)	**< 0.001**
*Tumor stage*			
pTa + pT1	28 (24)	27 (43)	**0.017**
≥ pT2	87 (76)	36 (57)	
*Histologic grade*			
G1	0	10 (14)	
G2	28 (24)	29 (46)	
G3	87 (76)	24 (40)	

* Reference 3.

### Comparable frequencies of TERT promoter mutations between Han Chinese and Western patients

To exclude the low *FGFR3* mutation observed in the present cohort of Chinese UCC patients due to technical problems such as lower sensitivity of Sanger sequencing, poor DNA quality, lower tumor cell numbers or others, we chose to use Sanger technology to screen the TERT promoter for mutation as a reference in these same cohorts of RPC and UBC patients, because the TERT promoter mutation has been shown to be widespread in UCC and closely associated with the *FGFR3* mutation. There are two hotspot mutations at the TERT proximal promoter region, namely C228T and C250T. These mutations were observed in 43% of RPC (38/91) and 52% UBC patients (60/116), respectively (Table 1 and 3, Figure 2). The presence of the TERT promoter mutation was not correlated with the *FGFR3* mutation either in RPC or UBC (Table 1 and 3, Figure 2).

## DISCUSSION

UCC is one of the most common malignancies worldwide [1, 3]. In Western countries, UBC accounts for approximately 95% of all UCCs whereas the RPC and UC incidence is low [1–3]. In contrast, there is a much higher prevalence of RPC and UC, although UBC remains predominant, in the Han Chinese population [7, 8]. The pathogenesis of UCCs is incompletely understood, whereas the accumulated evidence suggests that UCC initiation and progression is driven by an accumulation of genetic alterations, among which the *FGFR3* gene mutation occur most frequently, according to numerous clinical investigations obtained from Western/European patients [1, 3, 4, 14]. However, the present study shows that Han Chinese UCC patients have a very low frequency of the *FGFR3* mutation. More strikingly, the mutation was only found in 2.6% of UC patients, which is in sharp contrast to high as 54% of that in European patients reported by van Oer, et al. These results clearly reveal significant racial disparities in the genetic alterations between Han Chinese and Western UCCs. Similarly, different profiles of genetic mutations have also been observed in other types of human malignancies, for instance, there is a substantial difference in the *BRAF* mutation rate among white, black and Asian patients with colorectal cancer [23].

A key feature of the *FGFR3* mutation is its frequent occurrence in low stage/grade non-muscle-invasive UCC [1, 4, 14]. It is thus possible that the observed infrequent *FGFR3* mutation resulted from a stage bias in the present patient group. To exclude this, we searched for the published papers that investigated the *FGFR3* mutation in European RPC, UC and UBC simultaneously and found one report by van Oer et al. [3]. The comparison with van Oer's European cohort of patients clearly showed that the Han Chinese UBC patients had significantly lower rates of the *FGFR3* mutation despite a much higher percentage of them in pTa/T1 stages. A much higher *FGFR3* mutation was seen in European RPC and UC patients, but there was also a significant difference in stage/grade distributions between two cohorts of patients. Nevertheless, the stage/grade difference unlikely explains a low rate of the *FGFR3* mutation in Chinese patients, especially for UC, because even in the European patient group with ≥ pT2 stages, the mutation rate still remained 30 and 41% for RPC and UC, respectively [3], much higher than that seen in the present cohort of Chinese patients. During the preparation of the present manuscript, a newly published observation also shows a high frequency of the *FGFR3* mutation in American UTUC patients, especially those with high grades [21].

We also noticed the methodological difference in *FGFR3* mutation detection between us and van Oer et al. [3]: Sanger sequencing and SnaPshot were used in two studies, respectively, which raised a sensitivity issue. In addition, other technical biases such as tumor cell percentages and DNA quality might significantly affect accurate detection of the *FGFR3* mutation, too. Therefore, a good reference should be determined simultaneously. Recently, hotspot mutations of the TERT promoter were found to be widespread in UCCs including UBC, RPC and UC [5–8, 10]. Furthermore, the occurrence of TERT promoter and *FGFR3* mutations is highly correlated with each other, as shown in the studies of European patients (Allory et al., 2014; Hurst et al., 2014). In the present study, we did found a high rate of the TERT promoter mutation in the Chinese cohort of RPC (43%) and UBC (52%) patients, which is comparable with that in European patients. Such a high frequency of the TERT promoter mutation identified in these Chinese patients is in sharp contrast to a lack of the *FGFR3* mutation in the vast majority of them. It is thus evident that the *FGFR3* mutation is indeed an infrequent genetic event in Han Chinese UCC patients. The present data also suggest that the TERT promoter and *FGFR3* mutations may result from different mechanisms. Moreover, the racial disparities in the *FGFR3* mutation between Han Chinese and Western UCC indicate that genetic susceptibility and/or environment exposure could be different, which requires future investigations in details.

In the present cohort of UCC patients, the most frequent point mutation in the *FGFR3* gene is C746T (S249C), consistent with that observed in European patients [4, 14]. However, the presence of the *FGFR3* mutation was not associated with tumor stages or grades, and other clinical-pathological variables, likely due to a very low mutation rate. It is well established that the activating mutation of the *FGFR3* gene promotes urothelial cell proliferation and the MAPK signal pathway is one of the key the downstream effectors for the mutant FGFR3 [14]. If this gene mutation is rare in Han Chinese patients with UCC, what are alternative factors driving proliferation and oncogenesis of urothelial cells? The mutations of *H-* and *K-RAS* genes, also functioning via the MAPK signaling and mutually exclusive with the *FGFR3* mutation, occur in 10–15% of European UCCs [19, 24]. Gui et al. analyzed 97 Chinese UBC patients using whole-exome and/or Sanger sequencing and they observed comparable frequencies of the *RAS* gene mutations [25]. Conceivably, yet not characterized MAPK activators play parts in the pathogenesis of Han Chinese UCC. Whole genome and exome sequencing was performed on UBC tumors from Han Chinese patients [25, 26], however, there are no any clues about alternative driver mutations, which indicates that sequencing alone is insufficient to answer this question. Therefore, comprehensive analyses of whole genetic/epigenetic and proteomic landscapes in Han Chinese patients are required to solve this issue.

In summary, the results presented here demonstrate for the first time infrequent *FGFR3* mutations in Han Chinese patients with UCC including UBC, RPC and UC, which suggest racial disparities in the oncogenic pathway, genetic susceptibility and environment underlying the development of UCCs between Han Chinese and Western/European patients. The present findings also have important clinical implications: the *FGFR3* mutation is not a useful clinical biomarker for UCC diagnosis and prognosis in the Han Chinese population; targeting FGFR3 for Chinese UCC therapy may not be applicable in the vast majority of patients. Finally, our results underscore the need for extensive characterization of cancer genomes from diverse patient populations, thereby contributing to precision medicine for cancer treatment and prevention.

## PATIENTS AND METHODS

### Patients and tumor specimens

The study was conducted on the total of 363 Han Chinese UCC patients comprised of 138 with UBCs, 102 with RPCs and 123 with UC. The patients underwent surgery at Shandong University Qilu Hospital and Second Hospital, China. The specimens were collected after surgical treatment and kept frozen at −80°C or paraffin-embedded until use. All samples were collected with informed consent and the study was approved by the regional ethics committee (Shandong University Qilu Hospital and Second Hospital). The experiments were carried out in accordance with the approved regulations.

### DNA extraction and sequencing

Genomic DNA was extracted from frozen and/or paraffin-embedded tumor tissue samples using QIAGEN DNA extraction kits. DNA from tumour specimens was sequenced for the *FGFR3* gene mutation by Sanger sequencing and the specific PCR primers are as follow, as described [7]: Exon 7, 5′- AGT GGC GGT GGT GGT GAG GGA G-3′ (forward) and 5′- AGC ACC GCC GTC TGG TTG GC-3′ (reverse); Exon 10, 5′- CAA CGC CCA TGT CTT TGC AG-3′ (forward) and5′- CAA GAT CTC CCG CTT CCC G-3′ (reverse); and Exon 15, 5′-GAG AGG TGG AGA GGC TTC AG-3′ (forward) and 5′- TCA TGC CAG TAG GAC GCC T-3′ (reverse). To sequence the TERT promoter, we used primers and PCR conditions as described [27, 28]. The two mutations defined as C228T and C250T in the TERT core promoter correspond to positions 124 and 146 bp upstream of the ATG site. The PCR was performed with the following primer pairs: 5′-CACCCGTCCTGCCCCTTCACCTT-3′ (forward) and 5′-GGCTTCCCACGTGCGCAGCAGGA-3′ (reverse). All the mutations were verified by sequencing from both directions.

### Statistical analyses

Differences in the *FGFR3* mutation frequency between tumors with sex, clinical stage, and metastasis were determined using Fisher's exact test. All the tests were two-tailed and computed using SigmaStat3.1^®^ software (Systat Software, Inc., Richmond, CA). *P* values of < 0.05 were considered as statistically significant.
